# Inter-alpha-trypsin inhibitor heavy chain H3 is a potential biomarker for disease activity in myasthenia gravis

**DOI:** 10.1007/s00401-024-02754-6

**Published:** 2024-06-18

**Authors:** Christina B. Schroeter, Christopher Nelke, Frauke Stascheit, Niklas Huntemann, Corinna Preusse, Vera Dobelmann, Lukas Theissen, Marc Pawlitzki, Saskia Räuber, Alice Willison, Anna Vogelsang, Adela Della Marina, Hans-Peter Hartung, Nico Melzer, Felix F. Konen, Thomas Skripuletz, Andreas Hentschel, Simone König, Michaela Schweizer, Kai Stühler, Gereon Poschmann, Andreas Roos, Werner Stenzel, Andreas Meisel, Sven G. Meuth, Tobias Ruck

**Affiliations:** 1 Department of Neurology, Medical Faculty and University Hospital Düsseldorf, Heinrich Heine University, Moorenstr. 5, 40225 Düsseldorf, Germany; 2 Department of Neurology, Charité - Universitätsmedizin Berlin, 10117 Berlin, Germany; 3 Department of Neuropathology, Charité - Universitätsmedizin Berlin, Bonhoefferweg 3, 10117 Berlin, Germany; 4 Department of Neuropaediatrics, Neuromuscular Centre, Universitätsmedizin Essen, Hufelandstr. 55, 45122 Essen, Germany; 5 Brain and Mind Center, University of Sydney, 94 Mallett St, Sydney, Australia; 6 Department of Neurology, Palacky University Olomouc, Nová Ulice, 779 00 Olomouc, Czech Republic; 7 Department of Neurology, Hannover Medical School, 30625 Hannover, Germany; 8 Leibniz-Institut Für Analytische Wissenschaften – ISAS - E.V, 44227 Dortmund, Germany; 9 Core Unit Proteomics, Interdisciplinary Center for Clinical Research, Medical Faculty, University of Münster, 48149 Münster, Germany; 10 Electron Microscopy Unit, Center for Molecular Neurobiology Hamburg, University Medical Center Hamburg-Eppendorf, 20251 Hamburg, Germany; 11 Institute for Molecular Medicine, Proteome Research, University Hospital and Medical Faculty, Heinrich Heine University, 40225 Duesseldorf, Germany; 12 Molecular Proteomics Laboratory, Biological Medical Research Center, Heinrich Heine University, Universitätsstr 1, 40225 Duesseldorf, Germany

**Keywords:** Myasthenia gravis, Biomarker, Inter-alpha-trypsin inhibitor heavy chain H3, Muscle proteomics, Neuromuscular junction, Acetylcholine receptor

## Abstract

Myasthenia gravis is a chronic antibody-mediated autoimmune disease disrupting neuromuscular synaptic transmission. Informative biomarkers remain an unmet need to stratify patients with active disease requiring intensified monitoring and therapy; their identification is the primary objective of this study. We applied mass spectrometry-based proteomic serum profiling for biomarker discovery. We studied an exploration and a prospective validation cohort consisting of 114 and 140 anti-acetylcholine receptor antibody (AChR-Ab)-positive myasthenia gravis patients, respectively. For downstream analysis, we applied a machine learning approach. Protein expression levels were confirmed by ELISA and compared to other myasthenic cohorts, in addition to myositis and neuropathy patients. Anti-AChR-Ab levels were determined by a radio receptor assay. Immunohistochemistry and immunofluorescence of intercostal muscle biopsies were employed for validation in addition to interactome studies of inter-alpha-trypsin inhibitor heavy chain H3 (ITIH3). Machine learning identified ITIH3 as potential serum biomarker reflective of disease activity. Serum levels correlated with disease activity scores in the exploration and validation cohort and were confirmed by ELISA. Lack of correlation between anti-AChR-Ab levels and clinical scores underlined the need for biomarkers. In a subgroup analysis, ITIH3 was indicative of treatment responses. Immunostaining of muscle specimens from these patients demonstrated ITIH3 localization at the neuromuscular endplates in myasthenia gravis but not in controls, thus providing a structural equivalent for our serological findings. Immunoprecipitation of ITIH3 and subsequent proteomics lead to identification of its interaction partners playing crucial roles in neuromuscular transmission. This study provides data on ITIH3 as a potential pathophysiological-relevant biomarker of disease activity in myasthenia gravis. Future studies are required to facilitate translation into clinical practice.

**Supplementary Information:**

The online version contains supplementary material available at 10.1007/s00401-024-02754-6.

## Introduction

Myasthenia gravis (MG) is a chronic antibody (Ab)-mediated autoimmune disease impairing synaptic transmission at the neuromuscular junction (NMJ) [10, 42]. The clinical hallmark of MG is fatigable muscle weakness [11]. Approximately, 85% of cases are mediated by Abs directed against the acetylcholine receptor (AChR) [28].

Therapeutic decision-making relies mainly on clinical features, which are liable to fluctuation owing to factors, such as the time of day or effects of symptomatic medication. Previous studies suggested anti-AChR-Ab levels as potential biomarker reflecting the clinical course of disease. However, while routinely employed for diagnostic purposes, the predictive value of anti-AChR-Abs remains contested due to contradictory findings with some studies observing a correlation between Ab levels and clinical outcomes [18, 43], while others did not [6, 37]. This leaves a knowledge gap in the field of MG, as biomarkers indicating disease activity and identifying patients at risk for disease deterioration remain an unmet need, which is further intensified by the very recent advent of new therapeutic strategies in MG [15, 23, 42].

Here, we searched for such a serum biomarker as primary study outcome. Thus, we performed label-free mass spectrometry-based analysis of serum samples of two independent cohorts of anti-AChR-Ab-positive MG patients. We identified inter-alpha-trypsin inhibitor heavy chain H3 (ITIH3) as a potential biomarker for disease activity in MG using a machine learning (ML) algorithm.[3, 7, 8, 14, 26, 38] As previously reported, anti-AChR-Ab levels did not correlate with clinical outcomes in our cohort. This underscores the significant advantage offered by a novel and readily measurable serum biomarker.

## Materials and methods

### Ethics

The study was conducted in accordance with the Declaration of Helsinki and approved by the ethics committee of the Heinrich Heine University Duesseldorf and Charité Berlin (registration nos. 2021-1467, 2021-1417, 2021-1417_1, and 2021-1585 (Duesseldorf), EA1/281/10, and EA1/144/21 (Berlin)). All patients signed written informed consent before serum sampling.

### Patient recruitment and clinical data

Patients were recruited from two tertiary centers specialized in the management of MG (Heinrich Heine University Duesseldorf and Charité Berlin). 365 patients treated in the outpatient clinic or admitted to the hospital were asked for study inclusion. Out of 260 patients that agreed, 6 patients were lost to follow-up. The final cohort consisted of 254 MG patients. Patients were recruited from January 2016 to January 2022. Study inclusion required that patients met the national guidelines for MG diagnosis [48]: patients were required to have fatigable muscle weakness, evidence of anti-AChR-Ab and exclusion of potential differential diagnosis. At the time of serum sampling, all patients showed no evidence of infections following clinical and serological investigations. We included two cohorts of anti-AChR-Ab-positive MG patients (exploration and validation cohorts). Patient management was in accordance with the standards of the German Myasthenia Society [20, 28]. Patients were evaluated using the QMG and MG-ADL scores [2, 49]. The QMG score was assessed by a dedicated physician, while the MG-ADL score is patient reported using standardized forms.

Patient cohorts were assigned according to the following criteria:

- (1) Early and late onset: considering the results of the MGTX trial [47, 50], we defined late-onset MG as an initial diagnosis at the age of ≥ 60 years to reliably identify the changes of the proteome during the late stages of the disease.
- (2) IST: for the exploration and validation cohorts, patients were either treatment naïve or treated with one of the following drugs: prednisolone, azathioprine, methotrexate, or mycophenolate-mofetil, which was required to be at stable doses. Patients already receiving immunosuppressive therapy (IST) were required to not undergo a change of treatment during the 6-month period before the study baseline. A change of 5 mg over the screening period was permitted for prednisolone treatment. Patients with add-on therapies, such as efgartigimod, eculizumab, or ravulizumab, were excluded from these cohorts.
- (3) Active disease: MG patients with a QMG score $\le$ 7 were considered “PASS-positive,” i.e., in remission or minimal manifestation status, whereas patients with a QMG score > 7 were referred to as “PASS negative,” i.e., patients who do not consider their disease status as acceptable [24]. Similarly, an MG-ADL score ≤ 2 was considered “PASS positive” and > 2 as “PASS negative”.

For further comparative ELISA assays, we collected serum samples from 10 anti-muscle-specific kinase (MuSK)-Ab-positive and 10 seronegative MG patients, 14 patients with congenital myasthenic syndromes (CMS), 14 patients with idiopathic inflammatory myopathies (IIM), 19 patients with chronic inflammatory demyelinating polyradiculoneuropathy (CIDP), and 53 healthy controls (HCs).

### Biomaterial

All skeletal muscle specimens and serum samples had been cryopreserved at −80 °C prior to analysis according to the predefined standard operating procedure at the local biobank of the Heinrich Heine University Duesseldorf and Charité Berlin. For mass spectrometry-based analysis, serum samples were transferred on dry ice to the Core Unit Proteomics of the University of Münster.

### Lysate generation and processing for proteomic deep mapping

According to the manufacturer’s instructions, 200 µL of each serum sample was depleted using the ProteoMiner kit (Bio-Rad Laboratories Inc., Hercules, CA, USA) for comprehensive detection of proteins across the dynamic range of the proteome. This subproteome was placed in Pall Nanosep® 10 K Omega filter units (10 kDa cut-off; Pall, New York, USA) and centrifuged (12,500*g*, room temperature). The analyte was washed by adding 100 µL urea buffer (8 M urea, 100 mM Tris base) to the filter unit and centrifuging. For reduction (45 min), 100 µL 50 mM dithiothreitol in urea buffer was added to the filter unit. Subsequently, the unit was centrifuged again, and the sample was rinsed with 100 µL urea buffer. For alkylation, 50 mM iodoacetamide (IAA) in urea buffer was placed into the filter unit. Incubation proceeded in the dark for 30 min at room temperature. Following centrifugation and rinsing twice with 300 μL 50 mM NH_4_HCO_3_ containing 10% acetonitrile (ACN) in urea buffer, 200 µL 0.01 µg/µL trypsin in 50 mM NH_4_HCO_3_ containing 10% ACN was added to the filter unit. Incubation proceeded at 37 °C overnight. Peptides were collected by rinsing the filter thrice with 5% ACN/0.1% formic acid (FA), followed by centrifugation. Samples were dried using a Speedvac (Thermo Fisher Scientific, Waltham, MA, USA) and redissolved in 10 µL 5% ACN/ 0.1% FA.

### Mass spectrometry-based proteomics

Peptide solutions (0.5 µL) were analyzed by reversed-phase chromatography coupled to ion mobility mass spectrometry with Synapt G2 Si/M-Class nano-ultra performance liquid chromatography (UPLC) (Waters Corporation, Milford, MA, USA) using PharmaFluidics C18 µPAC columns (trapping and 50 cm analytical; PharmaFluidics, Ghent, Belgium), as described previously [4].

Data were analyzed using Progenesis for Proteomics (Waters) and the Uniprot human database. One missed cleavage was allowed, carbamidomethylation set as fixed, and methionine oxidation as the variable modification. A shortlist of the protein output was created by demanding protein assignment by at least two peptides, a fold value of at least 2, and ANOVA *p* ≤ 0.05. Quality controls (profile plots) were generated with Perseus v1.6.14.0. (see also Suppl. Figure 1, Online Resouce 1).

### Acetylcholine receptor autoantibodies (ARAb) radio receptor assay

Semi-quantitative determination of anti-acetylcholine receptor autoantibody levels in serum samples of the validation cohort was performed using a radio receptor assay kit (#RE21021, Tecan, Männedorf, Schweiz) according to the manufacturer’s instructions. Samples were measured in technical duplicates with the Tecan plate reader Infinite M200 Pro (Tecan).

### Enzyme-linked immunosorbent assay

Serum samples from patients and HCs were tested for ITIH3 abundance using an ITIH3 ELISA kit (#CSB-EL011896HU, CUSABIO, Houston, TX, USA) according to the manufacturer's instructions after the dilution series, and the samples were diluted 1:500 for the final assays. Samples were measured in technical duplicates with the Tecan plate reader Infinite M200 Pro (Tecan).

### Immunohistochemistry

All stains were performed on 8 µm cryostat sections, according to standard procedures. Immunohistochemical and double immunofluorescence reactions were carried out as described previously [13, 35]. We used irrelevant antibody stains (either mouse/rabbit monoclonal/polyclonal isotype controls) as negative controls, as well as omission of the primary antibody. The following antibodies were used for the staining procedures: C5b-9 (clone aE11, 1:200; DAKO, Ratingen, Germany), ITIH3 (PA5-22,232, polyclonal, 1:100; Thermo Fisher Scientific), and NSE (ab53025, polyclonal, and 1:100; Abcam, Cambridge, UK). The secondary antibodies were Goat Anti-Rabbit Alexa Fluor® 488, Goat Anti-Mouse Alexa Fluor® 488, Goat Anti-Mouse AF568, Goat Anti-Rabbit Cy3, or Goat anti-mouse Cy3 (all 1:100; Dianova, Hamburg, Germany). Gömöri trichrome staining was performed to stain collagen fibers for visualization of overall pathomorphology.

### ITIH3 protein precipitation from intercostal muscle

To further characterize the impact on muscular ITIH3 increase on a molecular level, utilizing the same primary anti-ITIH3-Ab as applied for immunostaining procedures, ITIH3 and interactors were precipitated from intercostal muscle (5 mg starting material) derived from two anti-AChR-Ab-positive MG patients, respectively, utilizing the “Pierce Protein A/G Magnetic Beads” kit (Thermo Fisher Scientific). Precipitations were performed according to the manufacturers’ instructions. To later exclude proteins non-specifically binding to the magnetic beads, for both intercostal muscles, an additional approach was carried out on whole protein extracts incubated with magnetic beads but without adding the primary anti-ITIH3-Ab. Given that our immunostaining studies excluded ITIH3 abundance in intercostal muscle derived from control individuals, sural nerve biopsies (5 mg starting material) derived from control cases were included as tissue controls.

#### Sample preparation for mass spectrometry

After elution, snap-frozen pull-downed protein complexes (ITIH3 interaction partners) were heated at 95 °C for 5 min and subsequently cooled to room temperature. Next, ice-cold acetone (in a threefold excess) was added, initiating protein precipitation at −20 °C overnight. Following this precipitation procedure, centrifugation at 12,000*g* at 4 °C for 20 min was performed to remove acetone. Afterward, the resulting protein pellets were air-dried under a fume hood to remove residual acetone. A solution of 8 M freshly prepared urea (50 µL) was added to dissolve the protein pellets.

Next, disulfide bonds were reduced by adding 10 mM TCEP at 37 °C for 30 min, while free sulfhydryl bonds were alkylated using 15 mM IAA at room temperature in the dark for 30 min. Subsequently, the sample solution was diluted to 1 M urea using 10 mM ABC (ammonium bicarbonate) buffer at pH 7.8. In-solution digestion was carried out using 1 µg trypsin (Sigma Gold), and samples were incubated at 37 °C overnight. After 15 h, the reaction was terminated by adding 2 µL of 99% FA. Solid-phase extraction with C18 filter cartridges (Waters) was employed for sample desalting, followed by washing with 0.1% trifluoroacetic acid (TFA) and elution with 80% ACN. The cleaned samples were dried using a vacuum concentrator and reconstituted in 20 µL of 0.1% TFA for mass spectrometric analyses.

#### LC–MS/MS analysis

Mass spectrometric analyses were conducted using an UltiMate 3000 RSLC nano UHPLC coupled to a Thermo Scientific LTQ Orbitrap Velos. Initially, samples were transferred to a 75 µm × 2 cm, 100 Å, C18 pre-column at a flow rate of 20 µL/min for 20 min. Subsequently, separation occurred on a 75 µm × 50 cm, 100 Å, C18 main column with a flow rate of 250 nl/min. A linear gradient was applied, consisting of solution A (99.9% water, 0.1% FA) and solution B (84% ACN, 15.9% water, 0.1% FA), with a pure gradient length of 100 min (3–38% Solution B). The gradient was executed as follows: 3% B for 20 min, 3–38% for 100 min, followed by three wash steps, each reaching 95% buffer B for 3 min. After the final wash step, the instrument equilibrated for 20 min at 3% buffer B. Data acquisition was performed in DDA data-dependent acquisition (DDA) mode.

The LTQ-Orbitrap Velos Pro instrument was operated in the DDA mode for automatic switching between full-scan MS and tandem mass spectrometry (MS/MS) acquisition. Single MS survey scans were conducted in the FTMS with a mass range of 300–2000 m/z, a resolution of 60,000, and a maximal ion injection time of 250 ms. The polysiloxane background ion (m/z 371.101236) was chosen as an internal mass for recalibrating the spectra in the Orbitrap.

A maximum of 10 MS/MS experiments were triggered per MS scan. The m/z values of signals already selected for MS/MS were added to an exclusion list for 30 s with an exclusion window size of ± 5 ppm. In all cases, a single microscan was recorded. Collision-induced dissociation (CID) was performed with a target value of 2000 ions in the linear ion trap, a normalized collision energy of 35%, a *Q*-value of 0.25, and an activation time of 10 ms.

#### Data analysis

For all data processing, the Proteome Discoverer software version 2.5.0.400 (Thermo Scientific, Schwerte, Germany) was employed. Searches were conducted in a target/decoy mode against a mouse UniProt database (downloaded on 30 November 2019, UniProt: www.uniprot.org) utilizing the MASCOT and SEAQUEST algorithms. The specified search parameters included precursor and fragment ion tolerances of 10 ppm and 0.5 Da for both MS and MS/MS analyses. Trypsin was designated as the enzyme with a maximum of two allowed missed cleavages. Carbamidomethylation of cysteine was set as the fixed modification, and the oxidation of methionine was defined as a dynamic modification. Additionally, a percolator false discovery rate was set to 0.01 on the PSM, peptide, and protein level.

An LFQ analysis was executed for each experimental condition. Proteins were considered significantly regulated with a *p* value of 0.05 or less after identification with at least two unique peptides and a ratio of 2 or higher (indicating a twofold enrichment) or a ratio of 0.5 or lower.

### Immunofluorescence

Double immunofluorescence stains were performed on 7 μm cryomicrotome sections of intercostal muscle biopsies of anti-AChR-Ab-positive MG patients as described previously [34]. In brief, slides were blocked with goat serum for 30 min at RT, followed by simultaneous application of the primary antibodies (information see below), overnight at 4 °C. After washing for 2 × 5 min in PBS, the corresponding secondary antibodies (goat anti-mouse AF488, goat anti-rabbit AF488, goat anti-mouse Cy3 or goat anti-rabbit Cy3, 1:100) were incubated for 1 h at RT. After a final washing step (2 × 5 min), slides were mounted with 4 ', 6-diamidino-2-phenylindole (DAPI)-containing medium and stored at 4 °C. The following antibodies were used for the staining procedures: (primary antibody, dilution, company/clone): rabbit anti-human ITIH3, 1:100, Abcam; mouse anti-human desmin, 1:100, Dako/D33; rabbit anti-human plectin, 1:100, Abcam/E398P; mouse anti-human CD56, 1:400, Serotec/ERIC-1.

### Visualization

Figures were created using Adobe Illustrator (version 2022).

### Statistical analysis

Statistical analysis was performed using *R* 3.5.3. Data were presented as median with IQR and mean ± standard deviation (SD), as absolute (*n*) or relative frequencies (%). Differences between groups were analyzed using an unpaired Student’s *t* test. The ANOVA test was used for multiple groups. To account for multiple comparisons, statistical significance was corrected by the false discovery rate (FDR) using a threshold of *Q* = 5%. Prior to multivariate analysis, data were centered, and unit variance scaling was used.

To identify differentially regulated protein subsets, overrepresentation analyses were performed using WEB-based GEne SeT AnaLysis Toolkit (WebGestalt at www.webgestalt.org/). For heatmaps, rows (representing proteins) and columns (representing patients) were clustered hierarchically using correlation distance and average linkage. Log_2_ mean expression values were color coded. MA plots were created by plotting the log_2_ fold change of protein intensity values between both depicted experimental groups (“M”) against their log_2_ mean expression levels (“A”). The significance level of differential protein expression profiles was labeled according to the following *p* values: *p* > 0.05 was classified as not significant, *p* ≤ 0.05 (*) as significant, *p* < 0.01 (**), *p* < 0.001 (***), and *p* < 0.0001 (****) as highly significant.

### Machine learning and predictor identification

ML was aimed at predicting the QMG score at the time of blood sampling as an outcome variable. The R package ‘caret’ (v6.0–88) was used as an ML pipeline. Briefly, our ML workflow is divided into data splitting, pre-processing, model training and tuning, and estimation of variable importance.

First, the data set was split 80:20 into a training data set used to build the final ML model and a test data set used for validation. This procedure ensures that the ML model cannot access data from the test set. Given a single class distribution of our data, data splitting was performed by random partitioning. Next, both data sets were pre-processed individually by centering and scaling the data. Because our ML model contains many potentially redundant features, data were cleansed by removing highly correlated predictors (cutoff value of *r* > 0.9 or *r* < −0.9) and zero- and near zero-variance predictors.

For ML training, we evaluated ML models capable of predicting a continuous, numerical outcome. To negate the potential risk of overfitting, we applied k-fold cross-validation (resampling) to our training data set. This method proposes dividing the initial data set into k groups, with k−1 groups used for ML training and the remaining group for in-group validation. The validation group is rotated k times, and the final accuracy is determined by computing the mean precision of the validation groups. tenfold cross-validation was applied [36].

ML algorithms were investigated by building models and evaluating their performance by application to the testing data. The lowest MAE was achieved for the final GLMboost model. Variable importance was extracted from the final model by computing the relative value of the t-statistic for each predictor variable. Importance was scaled between 0 and 100 based on the relative importance.

For simple regression analyses, we included the QMG and MG-ADL scores as dependent variables. For the evaluation of the predictive value of ITIH3 protein abundance for the course of the disease, regression analyses were repeated with ∆QMG and ∆MG-ADL scores after 12 months as dependent variables.

### Data transparency

Spectral raw proteome data were deposited in the PRIDE/ProteomeXchange repository (http://www.ebi.ac.uk/pride) and are available with identifier PXD040786.

Data from ITIH3 immunoprecipitation is depicted in Suppl. Table 1, Online Resource 2.

All data sets generated and analyzed during the current study, statistical analyses, and ML algorithms are available from the corresponding author upon reasonable request.

## Results

### Comprehensive proteomic mapping of serum samples

**Fig. 1:**
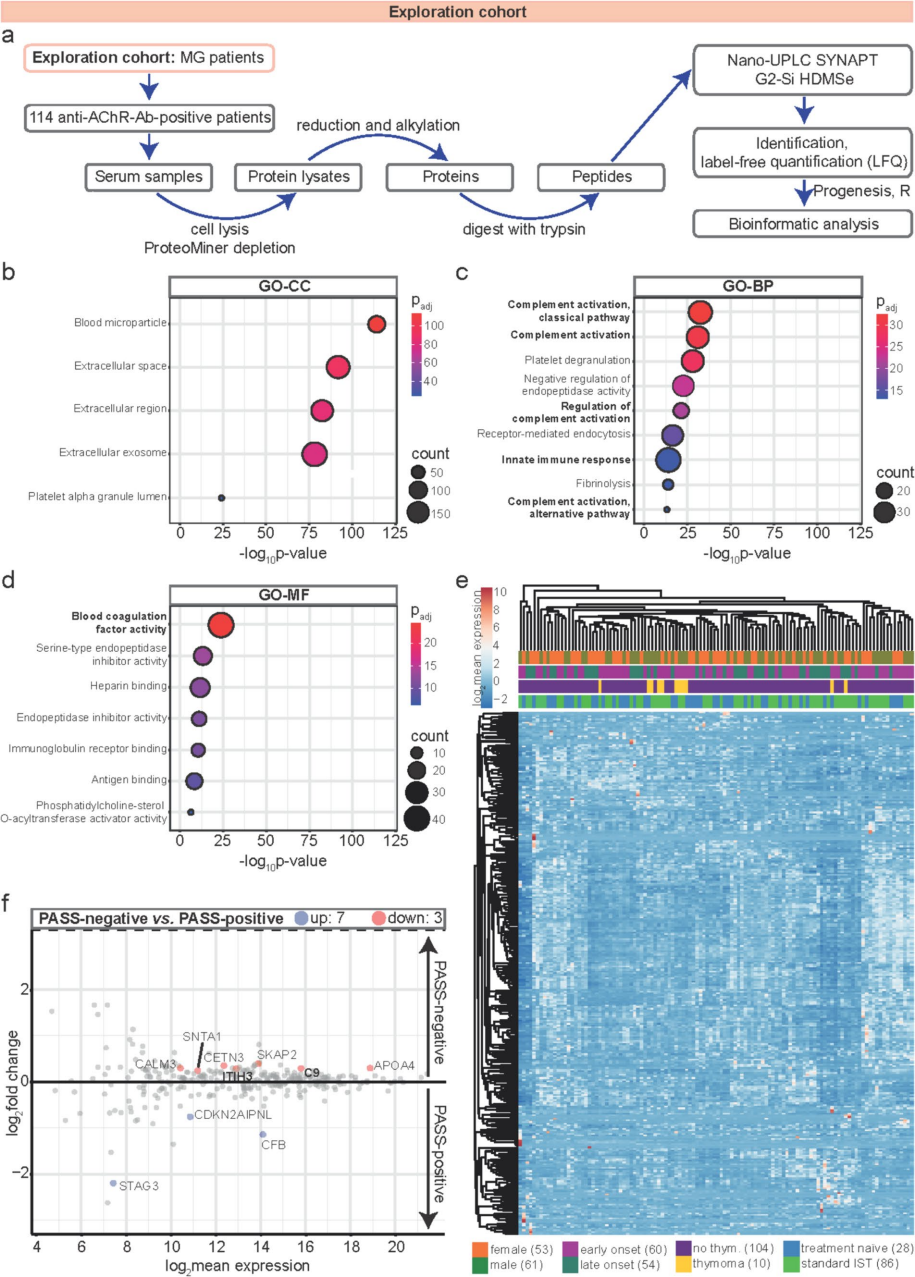
Comprehensive proteomic mapping of serum samples in the exploration cohort. **a** Workflow for mass spectrometry-based proteomics. Serum samples of 114 anti-AChR-Ab-positive MG patients were analyzed. Peptides were detected by nano-UPLC coupled to ion mobility MS with Synapt G2 Si/HDMSe. **b**–**d** ORAs of GO terms in the whole proteome. Negative decadic logarithms of corresponding *p* values (−log_10_ *p* value) are depicted on the x-axis. Circle sizes illustrate counts of associated proteins. P_adjs_ are color-coded. The most interesting terms referred to in the current study are printed in bold. **b** GO enrichment of cellular components (GO-CC). **c** GO enrichment of biological processes (GO-BP). **d** GO enrichment of molecular functions (GO-MF). **e** Hierarchical clustering of log_2_ mean expression levels of serum samples, i.e., patients (columns) and proteins (rows), according to Euclidean distance using correlation distance and average linkage. Log_2_ mean expression values are color-coded. Clinical subgroups of patients are depicted and illustrated in different colors. **f** MA plot illustrating differential protein expression profiles of PASS-negative versus PASS-positive patients by plotting the log_2_ fold change of protein intensity values against their log_2_ mean expression levels. All proteins with a *p* value of ≤ 0·05 were color-coded. The ten most significantly regulated proteins across both experimental groups were labeled with their gene symbols. *Anti-AChR-ab* anti-acetylcholine receptor antibody, *APOA4* apolipoprotein A4, *BP* biological process, *CALM3* calmodulin-3, *C9* complement component C9, *CC* cellular component, *CDKN2AIPNL* CDKN2AIP N-terminal-like protein, *CETN3* centrin-3, *CFB* complement factor B, *GO* gene ontology, *HDMSe* high-definition mass spectrometry, *IST* immunosuppressive therapy, *ITIH3* inter-alpha-trypsin inhibitor heavy chain 3, *LFQ* label-free quantification, *MF* molecular function, *MG* myasthenia gravis, *MS* mass spectrometry, *ORA overrepresentation analysis*, *P*_*adj*_ adjusted *p* value, *PASS* patient-acceptable symptom state, *SKAP2* src kinase-associated phosphoprotein 2, *SNTA1* alpha-1-syntrophin, *STAG3* cohesin subunit SA-3, *thym.* Thymoma, *UPLC* ultra performance liquid chromatography

We first recruited an exploration cohort of anti-AChR-Ab-positive MG patients (Fig. 1a) from a specialized center for MG (Berlin, Charité). Clinical data were acquired according to the standard procedure of the German Myasthenia Registry [27, 28]. All patients were followed for a minimum of 12 months. Visits were every 3–6 months with recording of clinical data including current treatment and quantitative MG (QMG) and MG activities of daily living (MG-ADL). Out of 120 patients, 114 patients completed the full observation period and were included in the final analysis, while six patients were lost to follow-up owing to a change of care provider. The clinical and demographic cohort data are shown in Table 1. As differences in treatment regimens are likely to represent a confounder, we aimed to study a homogenous cohort in respect to therapies. For this purpose, patients receiving add-on therapies at baseline, such as eculizumab, ravulizumab, and efgartigimod, were excluded and only standard IST was permitted.

**Table 1:** Clinical and demographic baseline data of the exploration cohort

Clinical characteristics	Prevalence (*n* = 114)
Anti-AChR-Ab positive, *n* (%)	114 (100%)
Gender, *n* (%)	
Female	53 (46.5%)
Male	61 (53.5%)
Age (years), median (IQR)	60 (30)
Onset, *n* (%)	
Early onset	60 (52.6%)
Late onset	54 (47.4%)
Thymoma, *n* (%)	
No thymoma	104 (91.2%)
Thymoma	10 (8.7%)
QMG score, median (IQR)	
Baseline^a^	6 (7)
After 12 months	5 (8.75)
MG-ADL score, median (IQR)	
Baseline^a^	3 (5)
After 12 months	3 (5.25)
Treatment, *n* (%)	
Treatment naïve	28 (24.6%)
Standard IST	86 (75.4%)
Prednisolone	5 (4.4%)
Azathioprine	69 (60.5%)
Methotrexate	7 (6.1%)
Mycophenolate-mofetil	5 (4.4%)
Prednisolone dose/day, median (IQR)	5 (8)

*AChR* acetylcholine receptor, *Ab* antibody, *IQR* interquartile range, *IST* immunosuppressive therapy, *MG-ADL* myasthenia gravis activities of daily living; no., number, *QMG* quantitative myasthenia gravis

^a^Baseline was defined as the time of blood sampling

All serum samples in our cohort were processed concurrently for mass spectrometric analysis. Altogether, 21,161 peptides were identified. Quality controls of the data set illustrated normal distribution of the proteomic data set without significant outliers (Suppl. Figure 1a, Online Resource 1). Gene ontology (GO) overrepresentation analysis of all quantified proteins revealed aberrant complement activation (Fig. 1b and c). Consistently, blood coagulation factor activity associated with immune homeostasis, [17, 21, 33] was the most enriched molecular function (Fig. 1d).

Taken together, the serum proteome of MG is characterized by aberrant complement pathways.

### Influence of disease activity on serum proteome

Next, we investigated the association between clinical features of interest and protein levels. The 114 protein profiles are shown as a heatmap with hierarchical clustering before downstream analysis (Fig. 1e).

Mendoza et al*.*[24] defined thresholds for commonly used MG health scales reflecting a patient-acceptable symptom state (PASS), i.e., patients in remission or minimal manifestation status. Accordingly, we used the proposed cutoff for the QMG score of > 7 for “PASS-negative” patients, i.e., patients with active disease state who did not consider their disease status acceptable. This cutoff score enables a binary grouping of patients based on a clinically relevant outcome. Proteins associated with the complement system were dysregulated in PASS-negative patients (Fig. 1f). As such, complement component C9 was increased in PASS-negative patients, while the levels of complement factor B (CFB) were decreased. Additionally, the level of ITIH3, belonging to the inter-alpha-trypsin inhibitor (IαI) family, was increased in PASS-negative patients. IαIs contain several complement-binding domains that inhibit the early phase of complement activation and thus mitigate complement-induced organ injury [9, 22, 31, 51].

In summary, active MG is characterized by a distinct set of proteins associated with complement pathways.

### ITIH3: a novel biomarker for MG disease activity and prognosis

**Fig. 2:**
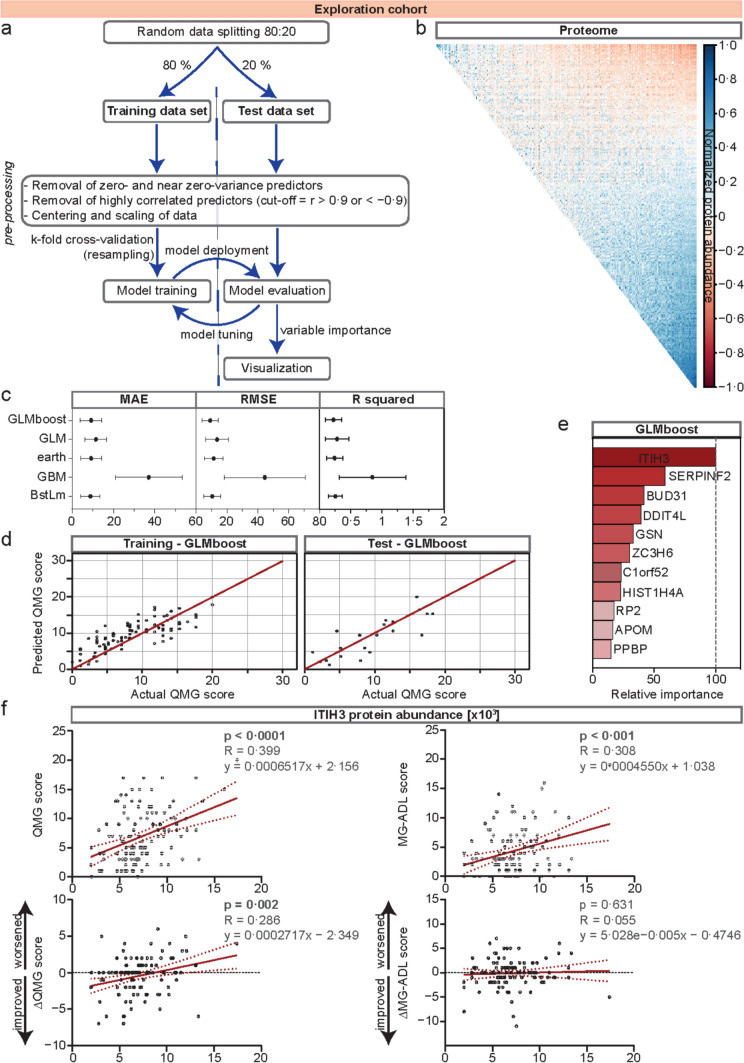
Machine learning identifies ITIH3 as a potential novel biomarker for disease severity and prognosis. **a** Workflow of the ML approach. Briefly, data were split into training and test data sets, followed by pre-processing of data, model training and tuning, and, finally, estimation of variable importance. **b** Correlation of a whole proteome data set. Highly correlated predictors with a cutoff value of *r* > 0·9 or < −0·9 were removed during pre-processing of data. **c** Comparison of five models: GLM, GLMboost, earth, GBM, and BstLm. Evaluation of these models comprises computing and comparing the MAE, the RMSE, and R-squared. Error values are indicated on the x-axis. **d** Actual versus predicted QMG scores for the final model (GLMboost). **e** The relative value of the t-statistic for each predictor variable is calculated and indicated in the bar graph. The highest contributing variable was scaled to 100. **f** Regression analyses of ITIH3 protein abundance and (∆)QMG or MG-ADL scores at the time of blood sampling (upper panels) or 12 months after first testing, respectively (lower panels). In the upper right hand of each plot, *p* values are indicated next to the R statistic and the linear function equation describing the regression. *APOM* apolipoprotein M, *BUD31* protein BUD31 homolog, *BstLm* boosted linear model, *C1orf52* UPF0690 protein C1orf52, *DDIT4L* DNA damage inducible transcript 4 like; earth, multivariate adaptive regression splines, *GBM* gradient-boosting machine, *GLM* generalized linear model, *GLMboost* gradient boosting with component-wise linear models, *GSN* gelsolin, *HIST1H4A* histone H4, *ITIH3* inter-alpha-trypsin inhibitor heavy chain 3, *MAE* mean absolute error, *MG-ADL* MG activities of daily living, *ML* machine learning, *PPBP* platelet basic protein, *QMG* quantitative MG, *RMSE* root mean square error, *RP2* protein XRP2, *SERPINF2*, alpha-2-antiplasmin, *ZC3H6* zinc finger CCCH domain-containing protein 6

Next, we were interested in the potential use of our proteome data set to identify biomarkers for relevant clinical readouts. We chose QMG score as primary outcome as this scale is a readily available clinical score, also used in current clinical trials and correlates with other common assessments including the MG-ADL and MG-QoL15 scale [2]. Given the data set complexity, we chose an ML algorithm for downstream processing. Briefly, our ML workflow consisted of a training (80% of data) and a test (20% of data) data set. This was followed by data pre-processing, model training, and tuning (for a detailed workflow, see Fig. 2a and b). The QMG score at the time of blood sampling was defined as the outcome variable. Finally, the model with the highest predictive power was selected and variable importance was estimated (Fig. 2c–e).

We evaluated five established ML models [3, 7, 8, 14, 26, 38], which are capable of predicting a continuous, numerical outcome by computing and comparing the mean absolute error (MAE), root mean square error (RMSE), and R-squared (Fig. 2c). The gradient boosting with component-wise linear model (GLMboost) performed best, as measured by the acquisition of the lowest MAE (Fig. 2c). To illustrate the performance of the GLMboost model to predict the QMG score, we displayed the actual versus predicted QMG scores for the training and test data sets (Fig. 2d). Given the initial data splitting, the final ML model had no access to the test data set prior to application of the final algorithm. Next, variable importance was extracted by computing the relative value of the t-statistic for each predictor variable (Fig. 2e). ITIH3 was the variable contributing the most to this model. Therefore, we investigated the predictive value of ITIH3 protein abundance for the QMG and secondarily the MG-ADL score in anti-AChR-Ab-positive MG patients. Indeed, regression analyses confirmed ITIH3 as a highly significant indicator for the QMG and MG-ADL scores at baseline (time of blood sampling) (Fig. 2f—upper panels). Finally, we were interested in the prognostic value of ITIH3 for disease activity. To this end, we calculated the changes to the QMG and MG-ADL scores (∆QMG and ∆MG-ADL) between baseline and 12-month follow-up. This difference was correlated with ITIH3 protein abundances (Fig. 2f—lower panels). The ITIH3 protein levels predicted the ∆QMG, but not the ∆MG-ADL score after 12 months (Fig. 2f).

Overall, combining mass spectrometry-based proteomics with ML identified ITIH3 as a potential biomarker with an indicative and prognostic value for MG.

### Validation in an independent cohort of MG patients

**Fig. 3:**
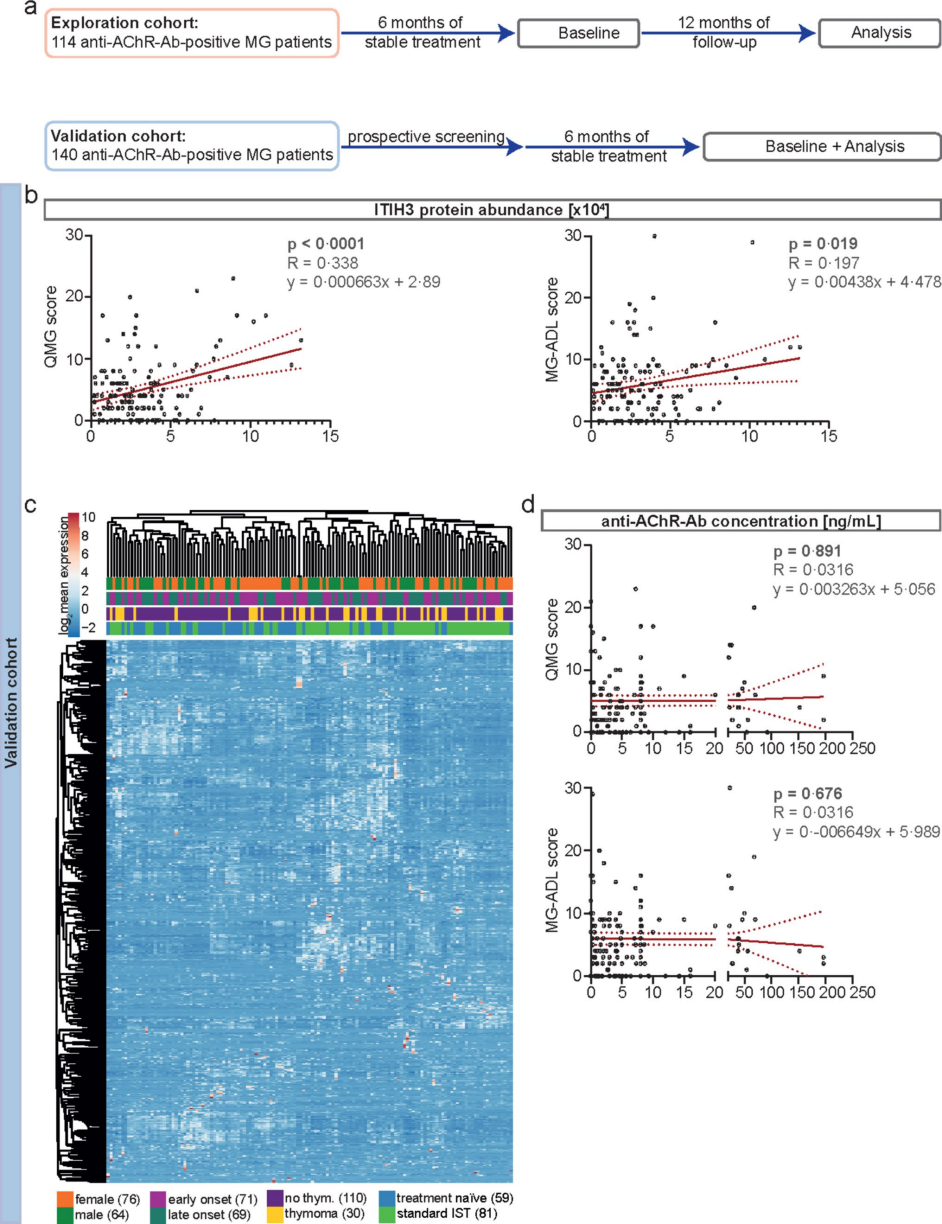
Validation of ITIH3 in an independent control cohort **a** Overview of the included patient cohorts. In the “exploration cohort,” the serum samples of 114 patients were collected at baseline, and patients were followed up for a minimum of 12 months. Patients receiving ISTs were required to be stable for at least six months before sample acquisition. For validation, 140 patients were recruited prospectively in the “validation cohort.” Blood samples were analyzed after collection. **b** Univariate regression analyses of ITIH3 protein abundance and QMG or MG-ADL scores at the time of blood sampling in the validation cohort. In the upper right hand of each plot, *p* values are indicated next to the R statistic and the linear function equation describing the regression. **c** Hierarchical clustering of log_2_ mean expression levels of serum samples in the validation cohort, i.e., patients (columns) and proteins (rows), according to Euclidean distance using correlation distance and average linkage. Log_2_ mean expression values are color-coded. Clinical subgroups of patients are depicted and illustrated by different colors. **d** Regression analyses of anti-acetylcholine receptor antibody levels and QMG or MG-ADL scores at the time of blood sampling in the validation cohort. The detection limit of the radio receptor assay used for this purpose was 0.4 ng/mL. In the upper right hand of each plot, *p* values are indicated next to the R statistic and the linear function equation describing the regression. *Anti-AChR-Ab* anti-acetylcholine receptor antibody, *IST* immunosuppressive therapy, *ITIH3* inter-alpha-trypsin inhibitor heavy chain 3, *MG* myasthenia gravis, *MG-ADL* MG activities of daily living, *QMG* quantitative MG, *thym.* thymoma

Given the large-scale data output of proteomic analysis, the risk of false positive results is increased. To address this, we prospectively recruited an independent validation cohort after completing our previous analyses. Here, serum samples were collected from 140 MG patients in a second specialized myasthenia center (Germany, Duesseldorf) (Fig. 3a). Clinical and demographic data are displayed in Table 2 and are similar to the exploration cohort; methodology for analysis was the same. Altogether, 21,292 peptides were identified. Quality controls of the data set illustrated normal distribution of the proteomic data set without significant outliers (Suppl. Figure 1b, Online Resource 1). We first fitted a linear regression model for ITIH3 protein abundance and the QMG and MG-ADL scores as readout parameters (Fig. 3b). Here, the ITIH3 protein levels correlated with the QMG and MG-ADL score at the time of blood sampling.

**Table 2:** Clinical and demographic baseline data of the validation cohort

Clinical characteristics	Prevalence (*n* = 140)
Anti-AChR-Ab-positive, *n* (%)	140 (100%)
Gender, *n* (%)	
Female	76 (54.3%)
Male	64 (45.7%)
Age (years), median (IQR)	57 (28)
Onset, *n* (%)	
Early onset	71 (50.7%)
Late onset	69 (49.3%)
Thymoma, *n* (%)	
No thymoma	110 (78.6%)
Thymoma	30 (21.4%)
QMG score, median (IQR)	
Baseline^a^	4 (5)
MG-ADL score, median (IQR)	
Baseline^a^	5 (6.25)
Treatment, *n* (%)	
Treatment naïve	58 (41.4%)
Standard IST	81 (57.9%)
Prednisolone	5 (3.6%)
Azathioprine	57 (40.1%)
Methotrexate	8 (5.7%)
Mycophenolate-mofetil	17 (12.1%)
Prednisolone dose/day, median (IQR)	6 (9)

*AChR* acetylcholine receptor, *Ab* antibody, *IQR* interquartile range, *IST* immunosuppressive therapy, *MG-ADL* myasthenia gravis activities of daily living; no., number, *QMG* quantitative myasthenia gravis

^a^Baseline is defined as the time of blood sampling

Next, we investigated whether ITIH3 concentration was influenced by clinical or demographic parameters other than disease activity. Therefore, we performed hierarchical clustering of all 140 protein profiles before downstream analysis and displayed the results in a heatmap without detecting clusters based on clinical metadata (Fig. 3c). We then combined the data sets from the exploration and validation cohorts and compared the ITIH3 distribution for sex, age at onset, thymoma status, and treatment status (Suppl. Figure 2a, Online Resource 1). Here, no statistically significant differences were detected among groups.

In a next step, due to ongoing discussions in the field, we determined the anti-AChR-Ab levels in all serum samples of the validation cohort using a semi-quantitative radio receptor assay. Regression analyses demonstrated no significant correlation between anti-AChR-Ab levels and the QMG and MG-ADL scores (Fig. 3d).

Taken together, we were able to validate ITIH3 as a biomarker for MG activity in an independent cohort. Lack of correlation between anti-AChR-Ab levels and clinical scores underlined the clinical need for new biomarkers indicating disease activity.

### Validation of ITIH3 as a biomarker for disease activity by immunoassays

**Fig. 4:**
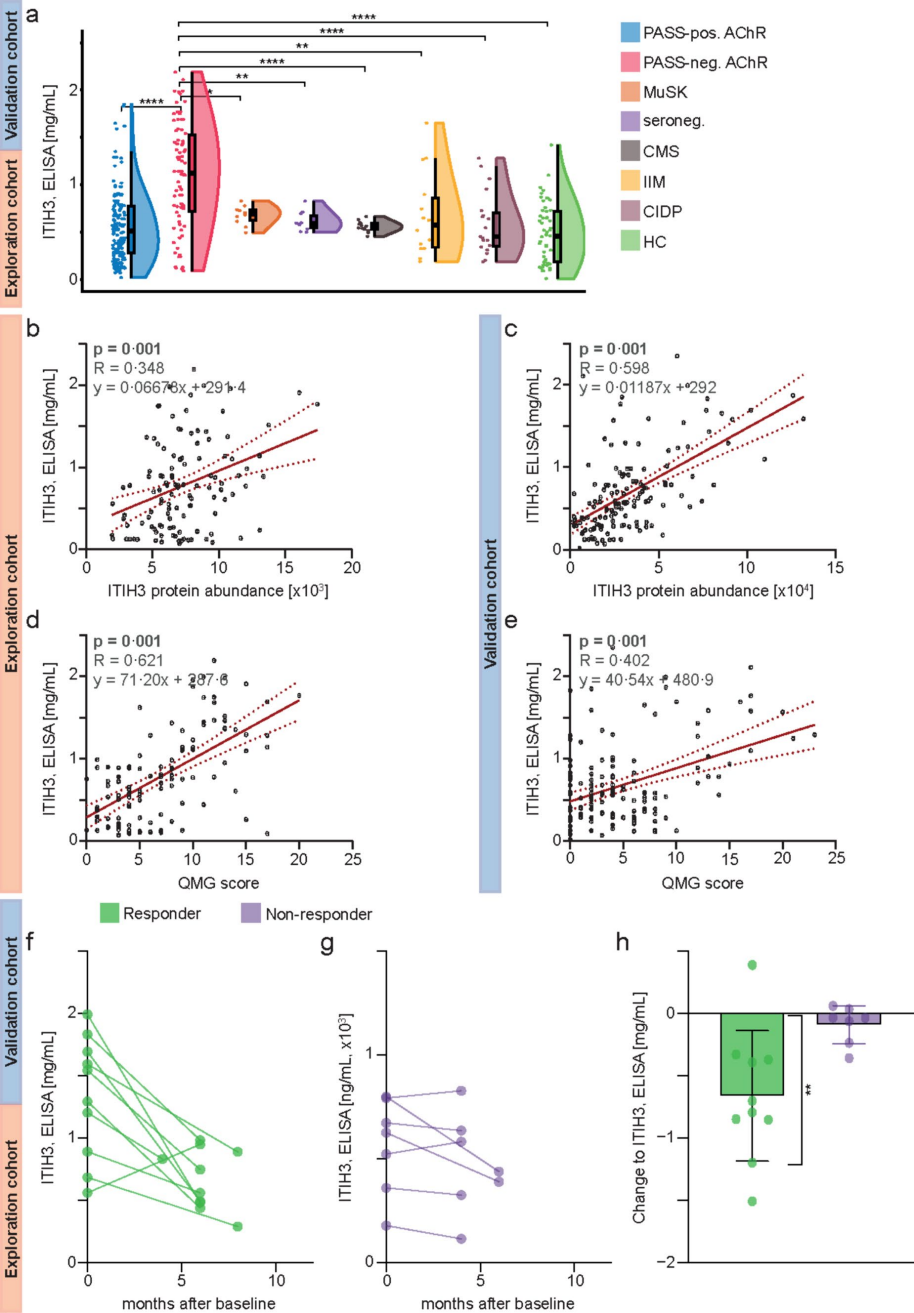
Validation of ITIH3 as a biomarker for disease activity and treatment response **a** Raincloud plot comparing ITIH3 protein abundance levels between PASS-positive and PASS-negative anti-AChR-Ab-positive MG patients measured by ELISA. Here, data sets from the exploration and validation cohorts were combined. Likewise, ITIH3 levels are indicated for anti-MuSK-Ab-positive (*n* = 10) and seronegative (*n* = 10) MG patients as well as for patients with CMS (*n* = 14), IIM (*n* = 12 with IBM and *n* = 2 with IMNM), and CIDP (*n* = 19). HCs indicate baseline levels (*n* = 53). A *p* value > 0.05 was classified as not significant, *p* < 0.05 (*) as significant, *p* < 0.01 (**), *p* < 0.001 (***), and *p* < 0.0001 (****) as highly significant. **b **+** c** Linear regression analysis of ITIH3 protein abundance measured by ELISA and by mass spectrometry in each cohort as indicated. **d **+ **e** Linear regression analysis of ITIH3 protein levels measured by ELISA and individual QMG scores in each cohort as indicated. In the upper left hand of the plot, *p*-values are indicated next to the R statistic and the linear function equation describing the regression. **f** Scatter plot displaying the change to serum ITIH3 as measured by ELISA for treatment responders between baseline and follow-up. Each line indicates the change for an individual patient. **g** Scatter plot displaying the change to serum ITIH3 as measured by ELISA for treatment non-responders between baseline and follow-up. Each line indicates the change for an individual patient. Treatment response was defined as an improvement of ≥ 3 points on the MG-ADL scale. **h** Bar graph indicating the change to ITIH3 between follow-up and baseline for responders and non-responders. A one sample *t* test with zero as hypothetical mean was used for statistical analysis of each group. A *p* value > 0.05 was classified as not significant, *p* < 0.05 (*) as significant, *p* < 0.01 (**), *p* < 0.001 (***), and *p* < 0.0001 (****) as highly significant. *Anti-AChR-Ab* anti-acetylcholine receptor antibody, *CIDP* chronic inflammatory demyelinating polyradiculoneuropathy, *CMS* congenital myasthenic syndrome, *HC* healthy control, *IBM* inclusion body myositis, *IIM* idiopathic inflammatory myopathy, *IMNM* immune-mediated necrotizing myopathy, *ITIH3* inter-alpha-trypsin inhibitor heavy chain H3, *MG* myasthenia gravis, *MuSK* muscle-specific kinase, *PASS* patient-acceptable symptom state, *seroneg.* seronegative, *QMG* quantitative MG

Next, we aimed to validate the potential value of ITIH3 to serve as a suitable biomarker making use of an alternative analytical approach. This strategy also aimed to demonstrate the robustness and analytical validity and to facilitate translation into clinical practice. Thus, we measured ITIH3 levels in 53 HCs. MG patients were stratified into PASS negative and PASS positive. ITIH3 serum levels were twofold higher in PASS-negative patients as compared to PASS-positive patients or HCs (Fig. 4a). To address the potential of ITIH3 serving as a biomarker enabling patient stratification, we next investigated whether increase in ITIH3 levels could also be observed in MG patients without anti-AChR-Abs, such as anti-MuSK-Ab-positive and seronegative MG patients (*n* = 10 each) (for further clinical and demographic baseline data, see Suppl. Table 1, Online Resource 1). We moreover compared our data with 14 patients (6 female, 8 male) harboring known pathogenic variants in CMS-genes (8 patients in *CHRNE*, 2 in *CHRNE* slow channel, 1 in *CHRNA1*, 1 in *CHRNB1*, 1 in *CHAT,* and 1 patient in *GFPT1*), with mean age 14 years (3–35 years; median age 13,5). Here, ITIH3 levels were not significantly elevated compared to HCs, but were similar to levels seen in PASS-positive anti-AChR-Ab-positive MG patients (Fig. 4a; Suppl. Table 2, Online Resource 1). We have also compared our results with ITIH3 levels in IIM (12 patients with inclusion body myositis (IBM) and 2 with immune-mediated necrotizing myopathy (IMNM)) and CIDP with a similar observation (Fig. 4a). Thus, ITIH3 serum levels in PASS-negative anti-AChR-Ab-positive MG patients were significantly elevated not only compared to PASS-positive patients of the same serogroup, but also compared to anti-MuSK-Ab-positive and seronegative MG, CMS, IIM, and CIDP patients.

Further, ITIH3 protein levels correlated between measurements by mass spectrometry and by ELISA in the exploration (Fig. 4b) and validation cohorts (Fig. 4c). In line, ITIH3 measured by ELISA correlated with the QMG and the MG-ADL score in the exploration (Fig. 4d; Suppl. Figure 2b, Online Resource 1) and validation cohorts (Fig. 4e; Suppl. Figure 2c, Online Resource 1).

Thus, ITIH3 provides value as biomarker reflecting disease activity and may be measured by different analytical approaches including ELISA, an important aspect in daily clinical routine.

### Longitudinal ITIH3 levels in response to treatment switch

Finally, we investigated a subgroup of 17 patients who were PASS negative based on their individual MG-ADL scores and were scheduled for receiving a treatment switch. For this purpose, we included patients from the exploration (*n* = 7) and validation (*n* = 10) cohort. Serum samples and clinical data including QMG and MG-ADL scores were acquired before and after initiation of treatment change. Individual data for each patient are given in Table 3. We chose the MG-ADL in this analysis, as treatment responders were defined by an MG-ADL improvement of ≥ 3 points in recent clinical trials [15]. After baseline, patients were observed for 4–8 months, and the individual follow-up time was defined as the period between baseline and the reappointment as scheduled for clinical routine. Concurrent treatment with steroids was required to be stable during the follow-up period. Eleven patients were treatment naïve at baseline, while 2 patients received azathioprine, 3 mycophenolate-mofetil and 1 rituximab. Based on clinical indication, patients were switched to different treatments, including azathioprine, methotrexate, efgartigimod, eculizumab, and rituximab. ITIH3 was measured at baseline and follow-up by ELISA. Individual ITIH3 levels are displayed for the responder (Fig. 4f) and non-responder (Fig. 4g) groups. Treatment responders had lower ITIH3 levels at follow-up, while ITIH3 was not significantly altered in non-responders (Fig. 4h).

**Table 3:** Clinical and demographic baseline data of follow-up patients

Age (years)	Gender	Baseline QMG	Baseline MG-ADL	Follow-up QMG	Follow-up MG-ADL	Baseline therapy	Follow-up therapy	Baseline ITIH3 (ng/ml)	Follow-up ITIH3 (ng/ml)	Follow-up time point (months)	Steroids (mg/day)	Responder
43	f	9	11	9	6	MMF	AZA	1592.849	890.391	8	0	Yes
68	m	13	8	13	5	Naïve	MTX	1203.973	832.151	4	5	Yes
54	m	10	25	4	8	Naïve	ECU	561.49	950.29	6	5	Yes
28	f	16	24	12	16	Naïve	EFG	1543.299	748.127	6	5	Yes
54	m	12	7	4	2	RTX	ECU	684.299	291.227	8	0	Yes
62	f	22	30	12	16	Naïve	ECU	1832.291	983.221	6	2.5	Yes
21	f	6	12	7	9	Naïve	AZA	890.829	560.322	6	5	Yes
32	f	12	18	8	12	Naïve	ECU	1993.289	482.991	6	0	Yes
68	m	20	19	2	3	AZA	ECU	1693.289	493.392	6	0	Yes
36	f	10	12	7	6	Naïve	EFG	1294.299	439.29	6	2.5	Yes
66	m	15	4	15	11	Naïve	AZA	792.382	827.382	4	5	No
61	m	2	3	2	3	MMF	AZA	178.299	114.293	4	7,5	no
73	m	10	4	10	3	MMF	AZA	672.407	635.495	4	0	no
37	f	1	3	1	2	Naïve	AZA	359.8676	325.841	4	0	no
31	f	13	11	13	12	Naïve	RTX	625.291	389.211	6	10	no
66	m	2	5	6	5	AZA	ECU	522.832	581.74	4	5	no
32	f	3	8	3	8	Naïve	AZA	798.299	438.128	6	0	no

Individual clinical and demographic data of follow-up patients. MG patients were defined as treatment responders if they improved by ≥ 3 points on the MG-ADL scale. ITIH3 levels were measured in serum samples by ELISA. All patients were anti-AChR-Ab positive

*AChR* acetylcholine receptor, *Ab* antibody, *AZA* azathioprine, *ECU* eculizumab, *EFG* efgartigimod, *f* female, *ITIH3* inter-alpha-trypsin inhibitor heavy chain H3, *m* male, *MG-ADL* myasthenia gravis activities of daily living, *MMF* mycophenolate-mofetil, *MTX* methotrexate, *QMG* quantitative myasthenia gravis, *RTX* rituximab

This longitudinal data provides a tentative link between change in disease activity following treatment switch and individual ITIH3 levels.

### Localization of ITIH3 at the NMJ

**Fig. 5:**
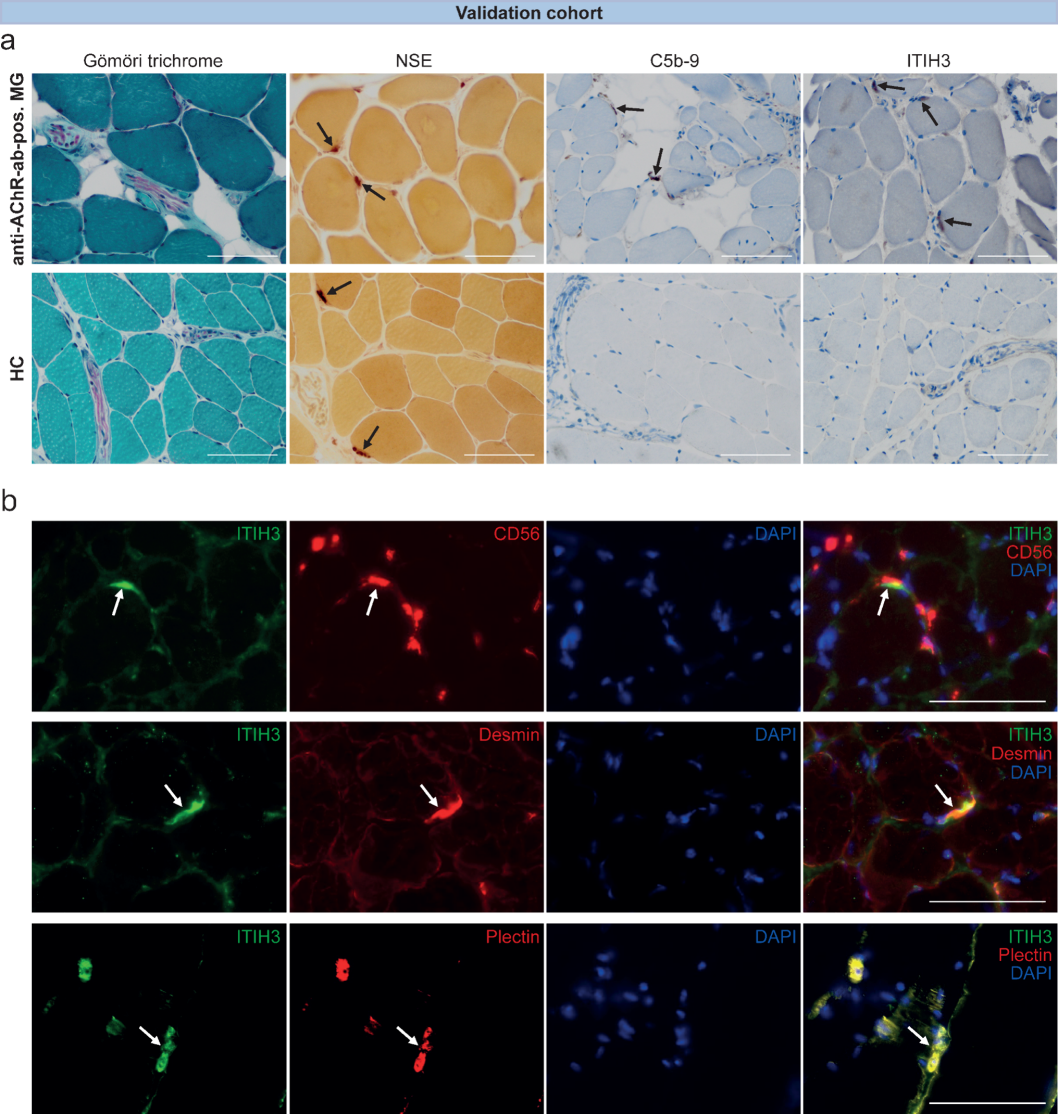
NMJs in skeletal muscles of anti-AChR-Ab-positive MG and healthy controls **a** Gömöri trichrome stain of skeletal muscle tissue showing mild fiber size variation and presence of endomysial nerve fascicles between myofibers (original magnification × 400). NSE indicating NMJs (arrows) within endplate regions both in skeletal muscle biopsies of anti-AChR-Ab-positive MG and HC (original magnification × 400). C5b-9 deposits at the NMJ in anti-AChR-ab-positive MG patients (arrows), but not in HC (magnification × 400). IHC for ITIH3 is positive at NMJ of anti-AChR-ab-positive MG (arrows), but not on endplates of healthy controls (note the proof of an endplate region by highlighting the nerve fascicle in the middle right) (original magnification × 400). The same exposure time was used for all images. Scale bars are 100 µm. **b** Double immunofluorescence staining of intercostal muscle biopsies from anti-AChR-Ab-positive MG patients (original magnification × 400). NMJs are indicated by arrows. The same exposure time was used for all images. Scale bars are 100 µm. CD56 is a neuronal cell adhesion molecule on the presynaptic membrane. Overlapping of CD56 and ITIH3 staining showed that ITIH3 is mainly localized at the postsynaptic membrane. Desmin is an intermediate filament-associated protein known to be enriched at the postsynaptic membrane of NMJs. Double immunofluorescence of ITIH3 and desmin showed an overlap of both markers, confirming the postsynaptic localization of ITIH3. Plectin is an intermediate filament-associated protein that links cytoskeletal components. It is highly expressed at the postsynaptic membrane and supports its junctional folds. Double staining of ITIH3 and plectin showed colocalization of both proteins. *Anti-AChR-Ab* anti-acetylcholine receptor antibody, *DDA* data-dependent acquisition, *DES* desmin, *HC* healthy control, *IHC* immunohistochemistry, *ITIH3* inter-alpha-trypsin inhibitor heavy chain H3, *LC* liquid chromatography, *MG* myasthenia gravis, *MS/MS* tandem mass spectrometry, *NMJ* neuromuscular junction, *NSE* neuron-specific enolase, *PLEC* plectin

We hypothesized that ITIH3 serum levels accumulate in response to tissue damage and complement activation at the NMJ. Thus, we also hypothesized that ITIH3 participates in the pathology at the NMJ. To address this assumption, we performed IHC of ITIH3 of anti-AChR-Ab-positive MG patients and HCs in intercostal muscle biopsies (Fig. 5a) [13]. Neuron-specific enolase (NSE) indicated neuromuscular endplates and C5b-9 complex (MAC) complement deposition at the NMJ in MG. Interestingly, we localized ITIH3 at the NMJ, which was highly up-regulated in MG compared to HCs.

To investigate the relevance of ITIH3 deposition to the NMJs and to identify interaction partners of ITIH3 and associated signaling pathways, immunoprecipitation studies were carried out making use of intercostal muscle biopsy specimens derived from patients with anti-AChR-Ab-positive MG. Mass spectrometry-based proteomic analysis was employed to identify 351 interaction partners in total, revealing striking candidates of functional relevance such as desmin and plectin as binding partners for ITIH3 in anti-AChR-Ab-positive MG (for detailed protein data see Suppl. Table 1, Online Resource 2).

To further validate this finding, we performed double immunofluorescence staining of intercostal muscle biopsies from anti-AChR-Ab-positive MG patients (Fig. 5b). First, we performed double staining for ITIH3 and CD56, a well-established neuronal cell adhesion marker of the presynaptic membrane [13], demonstrating a postsynaptic localization of ITIH3. A series of thoracic muscle biopsies (*n* = 9) from healthy control patients undergoing thoracic surgery for unrelated purposes were stained for ITIH3 and endplates were consistently negative, whereas they were physiologically CD56 positive. While desmin is an intermediate filament, plectin is an intermediate filament-associated protein. Both are known to stabilize the junctional folds at the postsynaptic membrane of neuromuscular endplates [5, 25, 39, 40]. In our study, colocalization of both desmin and plectin with ITIH3 confirmed the latter as a postsynaptic marker and strengthened the results of the previous immunoprecipitation analysis suggesting a role for ITIH3 in the stabilization of neuromuscular endplates.

Thus, we localized ITIH3 expression to the postsynaptic membrane of the NMJ in anti-AChR-Ab-positive MG and identified interaction partners of ITIH3, providing a structural basis and potentially underlying molecular pathways for our serological findings.

### Specificity of ITIH3 elevation for anti-AChR-Ab-positive MG patients

We moreover performed co-stainings of ITIH3 and C5b-9 as well as CD56 in muscle biopsy specimens derived from patients with DNA polymerase gamma deficiency (mitochondriopathy), rheumatoid arthritis and axonal polyneuropathy (neurogenic muscular atrophy), polymyalgia rheumatica (type 2B atrophy), and HCs (Suppl. Figure 3). We were able to identify NMJs in the depicted disease entities which is otherwise rare in limb skeletal muscles. Here, we observed that C5b-9 and ITIH3 stainings were negative in all four patient cohorts (Suppl. Figure 3A). Double immunofluorescence stainings of ITIH3 with CD56 as a presynaptic marker revealed that ITIH3 was negative in all four patient cohorts (except for faint non-specific staining of connective tissue structures), while presynaptic staining of endplates by CD56 was evident (Suppl. Figure 3B).

Therefore, we see these results as further evidence for the specificity of ITIH3 for anti-AchR-Ab-positive MG patients.

## Discussion

A deeper understanding of the mechanisms underlying MG pathogenesis is needed to improve disease management. Applying a proteomics approach to a large cohort of anti-AChR-Ab-positive MG patients, we report that (i) complement activation is a key driver of disease, as reported previously [13, 32], (ii) a distinct set of serum proteins characterize active disease, and (iii) in-depth characterization of those proteins by an ML approach identified ITIH3 as a potential biomarker for MG disease activity.

Regarding MG biomarkers, serum auto-Ab titers have been reported previously to correlate with disease activity [41, 44, 45] and/or treatment response [18, 43]. Specifically, anti-AChR-Abs were discussed to indicate disease activity; however, this observation was challenged by recent studies [6, 37]. This discrepancy from previous studies may be attributed to the heterogeneity of anti-AChR-Abs and the lack of standardized measurements [30, 43]. Further, differences in Ab pathogenicity may constitute a roadblock to the use of anti-AChR-Ab level as a biomarker for individual patients. In our study, anti-AChR-Ab levels did not correlate with clinical outcomes. While this observation has been contested in the past, our current data underline the need for novel biomarkers in the field given the tentative lack of a correlation between anti-AChR-ab levels and disease severity. Consequently, employing novel strategies, such as proteomic profiling, may help the search for candidate biomarkers in the field of MG.

Previous works identified ITIH3 and ITIH4—members of the IαI family—as inhibitors of early stages in the complement cascade, thereby reducing tissue injury [31]. In the current study, the ITIH3 protein levels were elevated in patients with active MG. Therefore, we hypothesize that ITIHs are enhanced in response to aberrant complement activation, thus serving as a negative feedback mechanism. Moreover, ITIHs bind to hyaluronic acid, a major extracellular matrix component [51] and thus contribute to extracellular matrix stabilization and tissue repair [1].

To select functionally relevant interactors, we made use of the recently published rare disease research workflow which also contains protein–protein interaction studies to elucidate the molecular etiology and disease severity in CMS as a genetic form of NMJ disease [29]. Of note, the multilayer communities of CMS-linked genes contain ITIH5, a further member of the IαI family, thus defining this as a suitable approach to decipher ITIH3 binding partners relevant for the function of the NMJ in the context of MG. Given that the largest module hereby displays a direct functional link between ITIH5 and plectin [29], a protein for which defects were linked to a treatment-responsive CMS subtype [12], plectin (which interlinks intermediate filaments with microtubules) was selected for further confirmational studies via immunostaining. Selection of desmin is based on its functional role as an intermediate filament in this context and a recent report on neuromuscular transmission defects caused by splice-site variants in the associated gene. This workflow combining immunoprecipitation with subsequent mass spectrometry enabled us to identify desmin and plectin as interaction partners of ITIH3 and confirmed their colocalization with ITIH3 at the postsynaptic membrane applying double immunofluorescence staining. Linkage of AChRs to postsynaptic desmin intermediate filament networks via plectin is crucial for postsynaptic NMJ organization [25]. Desmin may also be important for the organization of the postsynaptic cytoplasm being concentrated among and around the end of junctional folds [39]. Lack of desmin in mice leads to structural and functional disorders for the NMJ [5]. Plectinopathy (i.e., plectin deficiency or mutations) has been reported to cause myasthenic syndromes in patients [40]. Hence, interaction and colocalization of these proteins with ITIH3 in our data suggest that ITIH3 may stabilize the structural integrity of the neuromuscular endplate, in particular in the context of damage. However, we may have missed further interaction partners by this approach.

The diverse biological functions of ITIHs could explain their association with multiple conditions [46, 51]. As such, circulating ITIH3/4 levels are associated with carcinogenesis in colorectal cancer [16], and high ITIH4 levels correlate with a better prognosis in hepatocellular carcinoma [19]. Following this line of argumentation, ITIH3 might not provide diagnostic value to differentiate MG from a potential differential diagnosis, but instead serve as a biomarker in established disease. ITIH3 levels are neither elevated in other MG serogroups, such as anti-MuSK-Ab-positive or seronegative MG patients, nor in CMS. Also, ITIH3 levels are not increased in IIM as a muscle disorder and CIDP as a disease of the peripheral nerves. Further, this indicates a potential specific role of ITIH3 in complement-dependent destruction of the NMJ. Our cohorts demonstrate the applicability of ITIH3 as a biomarker in anti-AChR-Ab-positive MG as the largest subgroup. As illustrated in our subset of patients undergoing a treatment change, the measurement of ITIH3 serum levels could be beneficial for categorizing patients and predicting treatment outcomes.

A limitation to this study is potentially introduced by different treatment strategies. Identifying novel biomarkers requires the recruitment of an informative cohort that is both sizable and reflective of a general population of MG patients. The effect of individual ISTs is probably superimposed on individual protein signatures. Besides, there exists no general consensus currently on what constitutes active MG. We used a QMG score of > 7 to define active MG, because this cutoff score was used by Mendoza et al*.*[24] to determine patients who did not consider their disease status acceptable, thus defining patient-relevant outcomes. An advantage to this cutoff is its simplicity, enabling replication across different studies. In line, prospective studies are required to understand the potential value of ITIH3 for measuring disease activity and treatment responses in a longitudinal cohort.

In summary, this study provides the first data on ITIH3 as a potential biomarker for MG disease activity. Histological analyses provide a pathophysiological basis and validation across cohorts and techniques support the potential clinical applicability of our findings. The identification of the ITIH3 interactome is a first step toward understanding the underlying molecular mechanisms. However, future studies are required to validate ITIH3 and facilitate translation into clinical practice.

### Supplementary Information

Below is the link to the electronic supplementary material.

Supplementary file1 (DOCX 5134 KB)

Supplementary file2 (XLSX 105 KB)
