# Supplementary material for: Inter-alpha-trypsin inhibitor heavy chain H3 is a potential biomarker for disease activity in myasthenia gravis
Source: Acta Neuropathol. 2024 Jun 18;147(1):102. doi: 10.1007/s00401-024-02754-6 (PMC11195637; doi:10.1007/s00401-024-02754-6)
Supplement: Supplementary file 1 — Supplementary file1 (DOCX 5134 KB) [file 401_2024_2754_MOESM1_ESM.docx]

**Inter-alpha-trypsin inhibitor heavy chain H3 is a potential biomarker for disease activity in myasthenia gravis**

***Acta Neuropathologica***

Christina B. Schroeter, MD^1,†^; Christopher Nelke, MD^1,†^; Frauke Stascheit, MD^2^; Niklas Huntemann, MD^1^; Corinna Preusse, PhD^3^; Vera Dobelmann, M.Sc.^1^; Lukas Theissen, Cand.med.^1^; Marc Pawlitzki, MD^1^; Saskia Räuber, MD^1^; Alice Willison, MD^1^; Anna Vogelsang, PhD^1^; Adela Della Marina, MD^4^; Hans-Peter Hartung, MD^1,5,6^; Nico Melzer, MD^1^; Felix F. Konen, MD^7^; Thomas Skripuletz, MD^7^; Andreas Hentschel, PhD^8^; Simone König, PhD^9^; Michaela Schweizer, PhD^10^; Kai Stühler, PhD^11,12^; Gereon Poschmann, PhD^11^; Andreas Roos, PhD^1,4^; Werner Stenzel, MD^3^; Andreas Meisel, MD^2,†^; Sven G. Meuth, MD, PhD^1,†^; Tobias Ruck, MD^1,†^

**^†^These authors contributed equally to this work.**

**Author affiliations:**

1 Department of Neurology, Medical Faculty and University Hospital Düsseldorf, Heinrich Heine University Düsseldorf, Germany

2 Department of Neurology, Charité - Universitätsmedizin Berlin, 10117 Berlin, Germany

3 Department of Neuropathology, Charité - Universitätsmedizin Berlin, Bonhoefferweg 3, 10117 Berlin, Germany

4 Department of Neuropaediatrics, Neuromuscular Centre, Universitätsmedizin Essen, Hufelandstr. 55, 45122 Essen, Germany

5 Brain and Mind Center, University of Sydney, 94 Mallett St, Sydney, Australia

6 Department of Neurology, Palacky University Olomouc, Nová Ulice, 779 00 Olomouc, Czech Republic

7 Department of Neurology, Hannover Medical School, 30625 Hannover, Germany

8 Leibniz-Institut für Analytische Wissenschaften - ISAS - e.V, 44227 Dortmund, Germany

9 Core Unit Proteomics, Interdisciplinary Center for Clinical Research, Medical Faculty, University of Münster, 48149 Münster, Germany

10 Electron Microscopy Unit, Center for Molecular Neurobiology Hamburg, University Medical Center Hamburg-Eppendorf, 20251 Hamburg, Germany

11 Institute for Molecular Medicine, Proteome Research, University Hospital and Medical Faculty, Heinrich Heine University, 40225 Duesseldorf, Germany

12 Molecular Proteomics Laboratory, Biological Medical Research Center, Heinrich Heine University, Universitätsstr. 1, 40225 Duesseldorf, Germany

**Corresponding author:**

Tobias Ruck

Department of Neurology, Moorenstr. 5, 40225 Duesseldorf, Germany

Telephone: +49 211 81-19532

Fax: +49 211 81-18469

Email-address: Tobias.ruck@med.uni-duesseldorf.de

**Suppl. Table 1 Clinical and demographic baseline data for other MG serogroups**

| **Clinical characteristics** | **Anti-MuSK-Ab-positive MG** | **Seronegative MG** |
| --- | --- | --- |
| n | 10 | 10 |
| Gender, n (%) | | |
| Female | 7 (70%) | 7 (70%) |
| Male | 3 (30%) | 3 (30%) |
| Age (years), median (IQR) | 48 (50) | 51 (23) |
| Onset, n (%) | | |
| Early onset | 9 (90%) | 8 (80%) |
| Late onset | 1 (10%) | 2 (20%) |
| Thymoma, n (%) | | |
| No thymoma | NA | 7 (70%) |
| Thymoma | NA | 3 (30%) |
| QMG score, median (IQR) | | |
| Baseline^a^ | 7 (11.5) | 8.5 (13) |
| MG-ADL score, median (IQR) | | |
| Baseline^a^ | 5 (4.5) | 5 (8.05) |
| Treatment, n (%) | | |
| Treatment naïve | 0 (0%) | 3 (30%) |
| Standard IST | 10 (100%) | 7 (70%) |
| Prednisolone | 7 (70%) | 5 (50%) |
| Azathioprine | 3 (30%) | 5 (50%) |
| Methotrexate | 0 (0%) | 0 (0%) |
| Mycophenolate-mofetil | 3 (30%) | 0 (0%) |
| Rituximab | 2 (20%) | 0 (0%) |
| Prednisolone dose/day, median (IQR) | 15.0 (8.0-37.5) | 15.0 (7.0-21.5) |

^a^Baseline is defined as the time of blood sampling.

*Abbreviations: Ab, antibody; IQR, interquartile range; IST, immunosuppressive therapy; MG, myasthenia gravis; MG-ADL, myasthenia gravis activities of daily living; MuSK, muscle-specific kinase; n, number of replicates (here: patients); NA, not applicable; QMG, quantitative myasthenia gravis.*

**Suppl. Table 2 Descriptive statistics of ITIH3 ELISA levels across patient cohorts**

|  | **PASS-positive anti-AChR-Ab-positive MG** | **PASS-negative anti-AChR-Ab-positive MG** | **Anti-MuSK-Ab-positive MG** | **Seronegative MG** | **CMS** | **IIM** | **CIDP** | **HC** |
| --- | --- | --- | --- | --- | --- | --- | --- | --- |
| n | 176 | 78 | 10 | 10 | 14 | 14 | 19 | 53 |
| Median | 0.51 | 1.13 | 0.69 | 0.59 | 0.55 | 0.57 | 0.45 | 0.46 |
| IQR | 0.50 | 0.85 | 0.15 | 0.17 | 0.07 | 0.66 | 0.39 | 0.54 |
| Mean | 0.54 | 1.10 | 0.68 | 0.61 | 0.56 | 0.68 | 0.59 | 0.48 |
| SD | 0.36 | 0.56 | 0.10 | 0.10 | 0.05 | 0.45 | 0.33 | 0.31 |
| SEM | 0.03 | 0.06 | 0.03 | 0.03 | 0.01 | 0.12 | 0.08 | 0.04 |
| CV (%) | 66.48 | 50.63 | 15.01 | 16.84 | 9.80 | 65.91 | 56.10 | 65.06 |

*Abbreviations: Anti-AChR-Ab, anti-acetylcholine receptor antibody; CMS, congenital myasthenic syndrome; CV, coefficient of variation; HC, healthy control; ITIH3, inter-alpha-trypsin inhibitor heavy chain H3; IQR, interquartile range; MG, myasthenia gravis; MuSK, muscle-specific kinase; n, number of replicates (here: patients); PASS, patient-acceptable symptom state; SD, standard deviation; SEM, standard error of mean.*

# **Suppl. Fig. 1 Quality controls of the exploration and validation cohorts**

*
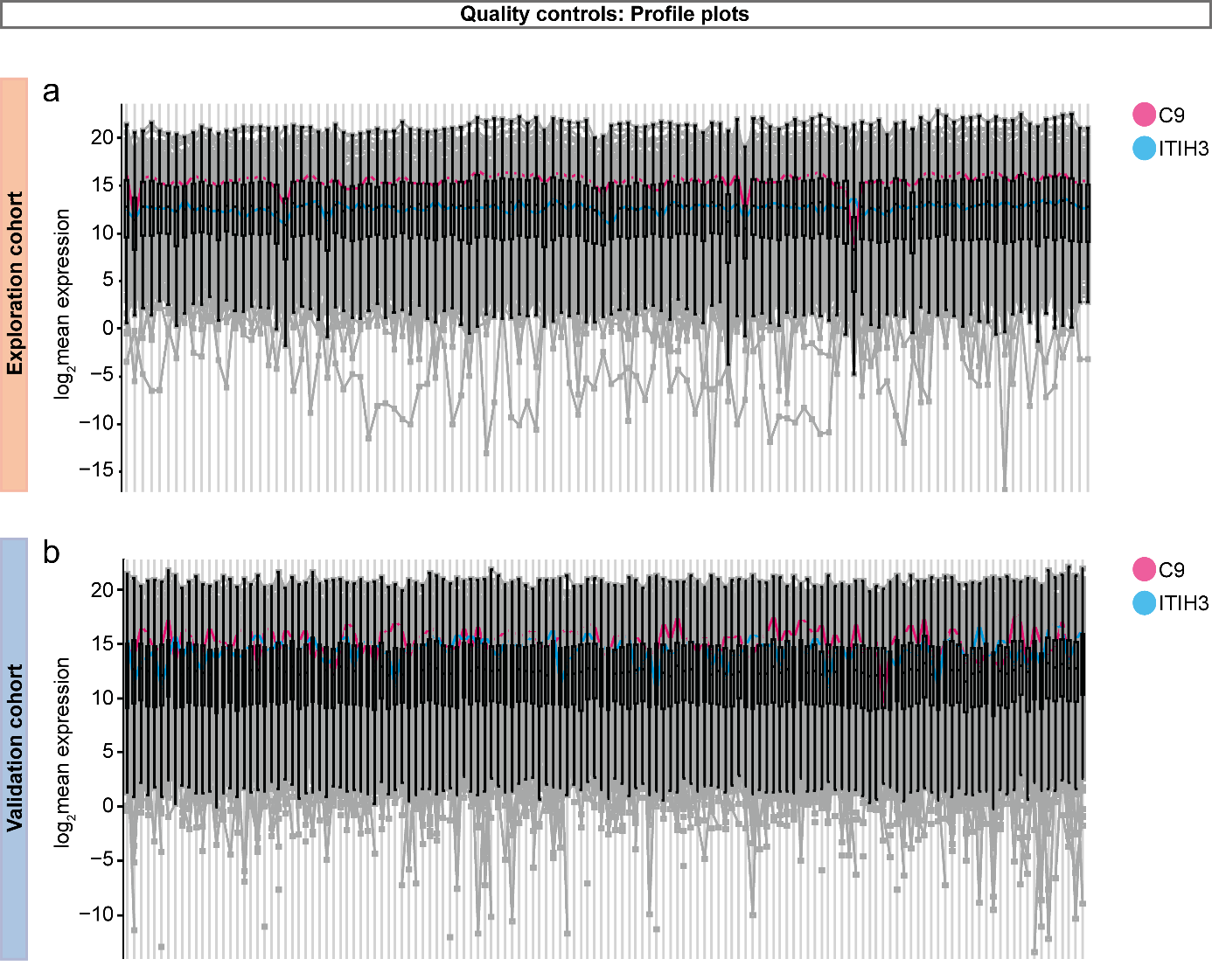
*Profile plots of the exploration **(a)** and validation **(b)** cohort display one quantitative profile for each protein, with each serum sample defining a data point, respectively. Boxplots show variance of all protein profiles across all runs. Profiles of C9 and ITIH3 were marked exemplarily.

*Abbreviations: C9, complement component C9; ITIH3, inter-alpha-trypsin inhibitor heavy chain H3.*

# **Suppl. Fig. 2 ITIH3 distribution across clinical subgroups in both the exploration and validation cohorts**

**
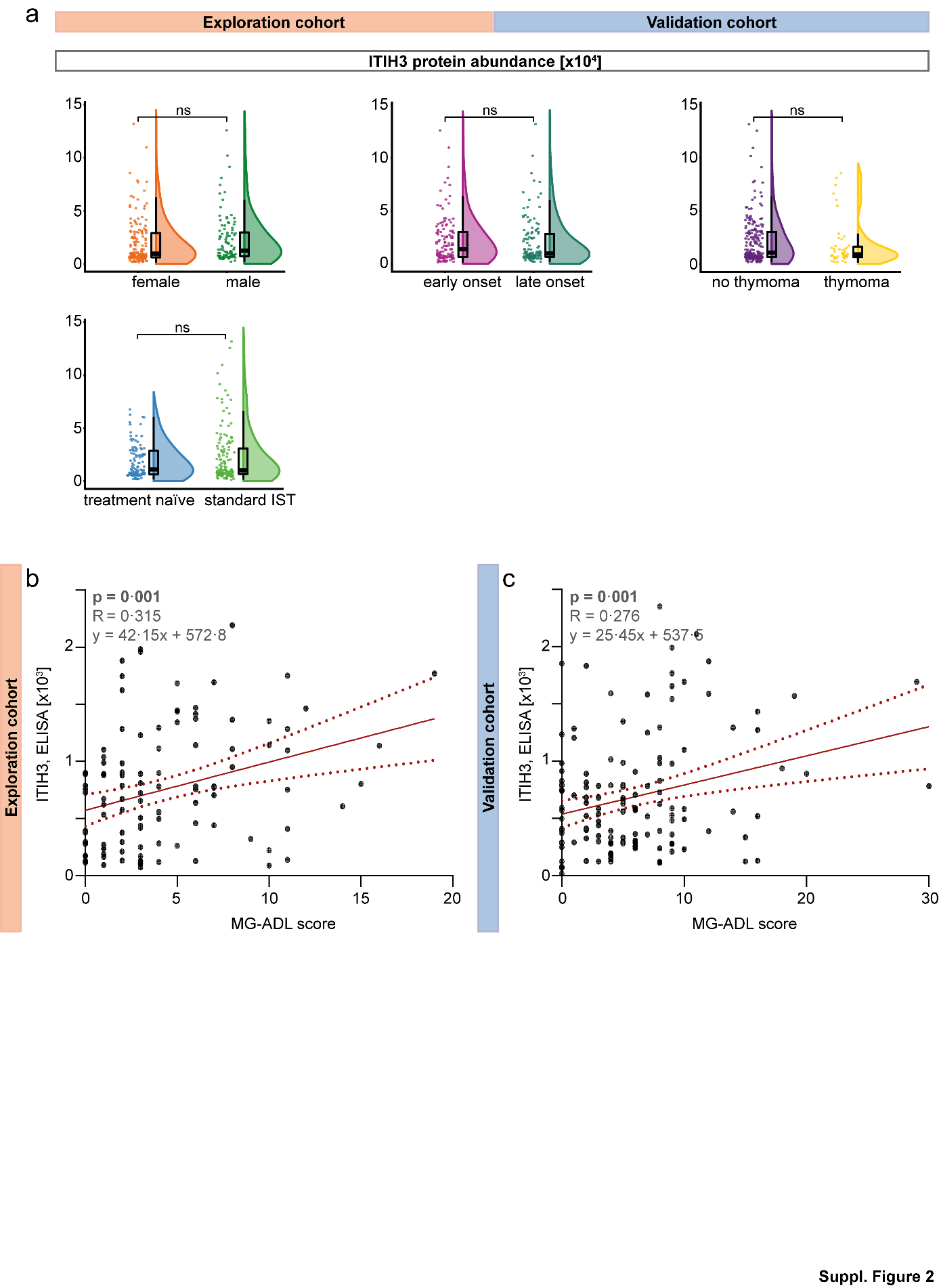
 (a)** Raincloud plots displaying ITIH3 distribution across clinical subgroups. Here, the datasets from the exploration and validation cohorts were combined. **(b + c)** Univariate regression analyses of ITIH3 protein abundance measured by ELISA and the MG-ADL scores in the exploration and validation cohorts. In the upper left-hand of each plot, p-values are indicated next to the R statistic and the linear function equation describing the regression. A p-value > 0.05 was classified as not significant, p < 0.05 (*) as significant, p < 0.01 (**), p < 0.001 (***), and p < 0.0001 (****) as highly significant.

*Abbreviations: ELISA, enzyme-linked immunosorbent assay; IST, immunosuppressive therapy; ITIH3, inter-alpha-trypsin inhibitor heavy chain H3; MG, myasthenia gravis; MG-ADL, MG activities of daily living; ns, not significant.*

**Suppl. Fig. 3 Immunohistochemistry and double immunofluorescence staining of muscle bi-opsies from patients with other muscular disorders and healthy controls.** *
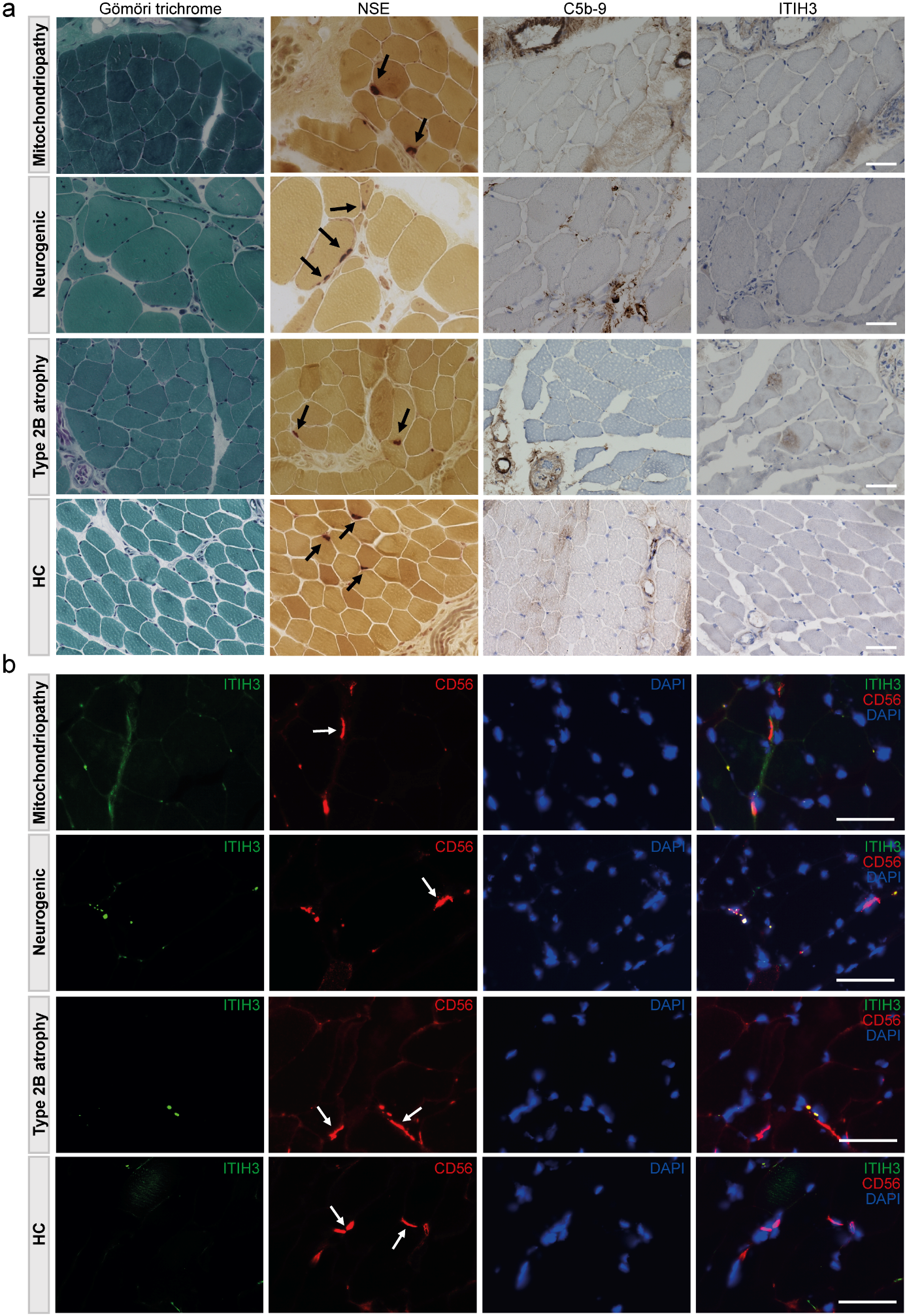
*

**(a)** Gömöri trichrome stain of skeletal muscle tissue from patients with DNA polymerase gamma defi-ciency (mitochondriopathy), rheumatoid arthritis and axonal polyneuropathy (neurogenic muscular atrophy), polymyalgia rheumatica (type 2B atrophy), and HCs showing mild fibre size variation and presence of endomysial nerve fascicles in between myofibers. NSE indicating NMJs (arrows) within endplate regions. IHC for C5b-9 deposits and ITIH3 is negative at NMJs of all shown disease entities and HCs. The same exposure time was used for all images. Original magnification x40, scale bars are 50 µm. **(b)** Double staining of CD56 with ITIH3 demonstrating that ITIH3 is absent at the NMJs of patients with other muscular disorders and HCs. CD56 is a neuronal cell adhesion molecule on the presynaptic membrane. NMJs are indicated by arrows. The same exposure time was used for all images. Original magnification x20, scale bars are 50 µm.

*Abbreviations: Anti-AChR-Ab, anti-acetylcholine receptor antibody; HC, healthy control; IHC, im-munohistochemistry; ITIH3, inter-alpha-trypsin inhibitor heavy chain H3; MG, myasthenia gravis; NMJ, neuromuscular junction; NSE, neuron-specific enolase.*
